# Renal sympathetic denervation in patients with vasospastic angina

**DOI:** 10.1007/s12350-019-01598-y

**Published:** 2019-02-13

**Authors:** Lida Feyz, Maureen Henneman, Fred Verzijlbergen, Isabella Kardys, Nicolas M. Van Mieghem, Joost Daemen

**Affiliations:** 1 Department of Cardiology, Thoraxcenter, Erasmus Medical Center, P.O. Box 2040, 3000 CA Rotterdam, The Netherlands; 2 Department of Radiology and Nuclear Medicine, Erasmus Medical Center, Rotterdam, The Netherlands; 3 Present Address: Department of Radiology and Nuclear Medicine, Radboud University Medical Center, Nijmegen, The Netherlands

**Keywords:** Renal sympathetic denervation, vasospastic angina, MIBG, meta-iodobenzylguanidine, quality of life

## Abstract

**Background:**

Sympathetic overactivity has been linked to vasospastic angina (VSA), although the exact pathophysiology of VSA is poorly understood. The purpose of this study is to assess if renal sympathetic denervation (RDN) reduces cardiac sympathetic nerve activity with a subsequent beneficial effect on angina relief in patients with refractory VSA.

**Methods and results:**

Cardiac sympathetic nerve activity was assessed prior to procedure and at 6 months post-procedure using iodine-123 labeled meta-iodobenzylguanidine (^123^I-MIBG) imaging. The Seattle Angina questionnaire (SAQ) was used to assess the degree to which the disease impacts quality of life. No significant change was observed in early HMR (pre-RDN: 2.74 [2.10 to 3.21] vs 6 months post-RDN: 2.57 [2.20 to 3.00]; *P* = 0.76), and late HMR (pre-RDN: 2.56 [2.18 to 3.20] vs 6 months post-RDN: 2.36 [2.13 to 3.22]; *P* = 0.22). Additionally, no change was seen in WR (*P* = 0.22). SAQ results revealed significant improvements in perceived physical limitation, angina frequency, and quality of life at 6 months (*P* < 0.05 for all).

**Conclusion:**

RDN resulted in improvements in angina class and quality of life at 6 months in patients with refractory VSA. RDN, however, did not result in significant changes in cardiac sympathetic nerve activity as measured using ^123^I-MIBG. The latter observation should be considered with caution given the small sample size of this study. Larger studies are needed to assess this further.

**Electronic supplementary material:**

The online version of this article (10.1007/s12350-019-01598-y) contains supplementary material, which is available to authorized users.

## Background

Vasospastic angina (VSA) is a clinical syndrome that was first described by Prinzmetal et al. in 1959.^1^ Although the syndrome has been characterized by episodes of coronary artery vasospasm, the exact pathophysiology of spasms is poorly understood. While studies from the early ‘80s and ‘90s already demonstrated that both the sympathetic and parasympathetic nervous system are responsible for coronary vasomotion, more recent work demonstrated significant hyperactivity of the sympathetic nervous system (SNS) in patients with VSA as compared to healthy controls.^2^–^4^ The majority of VSA patients present with refractory angina; however, the disease appears not benign. Syncope and ventricular arrhythmias are the first clinical presentation in up to 40% of the cases and the risk for sudden death may be increased by up to 50%.^5^–^8^ Current treatment options are mainly pharmacological and include the use of long-acting nitrates and calcium channel blockers, which showed to provide symptom improvement in 30 to 80% of the cases, respectively.^9^ Looking for more effective treatments, several small studies showed that surgical (cardiac) sympathetic denervation resulted in a significantly lower number of angina episodes and ST-segment deviations on 24h holter monitoring.^10^,^11^ In the present study, we hypothesized that renal sympathetic denervation (RDN), a percutaneous treatment that proved to significantly lower blood pressure and systemic sympathetic nerve activity in hypertensive patients, might provide additional angina relief in patients with VSA. In order to quantify the potential effect of RDN on cardiac sympathetic nerve activity, iodine-123-labeled meta-iodobenzylguanidine (^123^I-MIBG) scintigraphy was used.^12^,^13^

## Methods

### Study Population and Endpoints

Between April 2013 and August 2016, a total of 10 consecutive patients with refractory VSA underwent RDN. Patients were eligible in case of refractory angina. Significant residual coronary artery stenosis was ruled out and patency of previously implanted stents was confirmed in all cases by recent coronary angiography. Coronary physiologic assessment using fractional flow reserve was used in case of intermediate lesions.

VSA was confirmed in case of spontaneous or methylergonovine-induced spasms with ST-segment changes and symptoms of chest pain in 6 out of 10 patients. In 4 patients, the diagnosis was based on the presence of recurrent episodes of non-exercise-induced angina, which resolved after sublingual nitrates and in the absence of significant atherosclerotic coronary artery disease.

Patients were screened for RDN and followed according to routine clinical practice. Work-up included 24h ambulatory blood pressure measurement (24h ABPM), laboratory analysis, echocardiography, and CT (N = 2), MRI (N = 7) or renal duplex (N = 1) to confirm renal artery eligibility.

Follow-up in the outpatient clinic at 1, 3, and 6 months post-RDN included 24h ABPM (at 3 and 6 months) and echocardiography (at 6 months). Renal function was assessed at baseline and at follow-up. Renal artery imaging was performed at 6 months to confirm renal artery patency.

For the purpose of this, study patients were not subjects to acts, neither was any mode of behavior imposed, otherwise than as part of their regular treatment. Therefore, according to Dutch law, written informed consent for study enrolment was obtained. This study was conducted according to the privacy policy of the Erasmus Medical Center and the Erasmus Medical Center regulations for the appropriate use of data in patient-orientated research, which are based on international regulations, including the Declaration of Helsinki. All patients consented to the use of their data for scientific research.

The primary efficacy endpoint was the change in cardiac sympathetic nerve activity as measured using ^123^I-MIBG imaging at 6 months post-RDN, as compared to pre-RDN (baseline). The primary safety endpoint was defined as the occurrence of cardiovascular death, stroke, major access site bleeding, and acute kidney injury or renal artery stenosis at 6-month follow-up.

Secondary endpoints included the changes in Canadian Cardiovascular Society grading (CCS class), blood pressure (24h ABPM and office blood pressure), and heart rate. The Seattle Angina Questionnaire (SAQ) was used to assess the quality of life and signs and symptoms of angina.

### ^123^I-MIBG Scintigraphy Data Acquisition and Analysis

**Figure 1:**
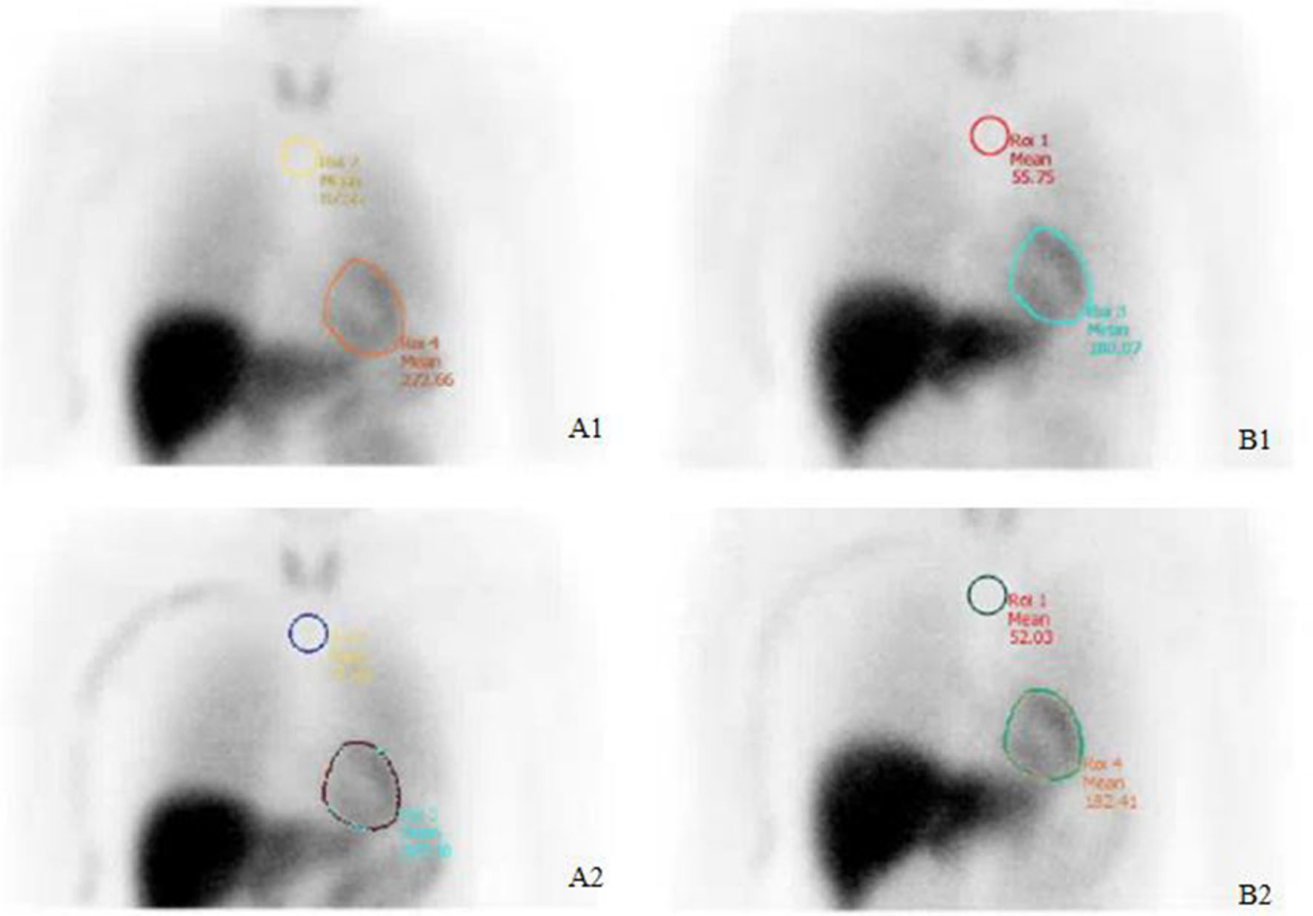
Example of MIBG image scoring with early and late HMR. Early HMR at baseline (A1) was 3.11 and changed to 3.35 at 6 months (A2), while late HMR at baseline was 3.23 (B1) and changed to 3.51 at 6-month (B2) follow-up. (**A**) 15 min after tracer injection, (**B**) 4 h after tracer injection

^123^I-MIBG is a physiologic analog of norepinephrine and acts selectively on sympathetic nerve endings. By using cardiac neurotransmission imaging, global information about neuronal function can be expressed in early, but more specifically in late HMR (reflecting the storage regional distribution and release of ^123^I-MIBG), with washout rate (WR) reflecting the neuronal integrity or sympathetic tone.^14^ In order to block thyroid uptake of free radioactive iodide, 200 mg potassium iodide (10% solution) was administered. After 30 minutes, 185 MBq ^123^I-MIBG was administered intravenously. Early and late (i.e., 15 minutes and 4 hours after tracer injection, respectively) anterior planar scintigraphic images were acquired for 10 minutes with a zoom factor of 1.0 and stored in a 256 × 256 matrix. Patients were imaged in a supine position with a dual head gamma camera (Symbia T, Siemens, Erlangen, Germany), using a medium energy collimator. An energy window of ± 10% was symmetrically centered around the 159-KeV 123I photo peak. Offline processing software (Hermes Medical Solutions Workstation) was used to draw a round region of interest (ROI) with fixed diameter over the upper mediastinum, below the thyroid gland (Figure 1). Additionally, a manual ROI over the heart was drawn, carefully excluding adjacent activity in the liver and lung. The left ventricular cavity was included in the myocardial ROI. The MIBG images were scored by a dedicated nuclear medicine specialist blinded to the timing of the scan. The HMR was computed by dividing the average number of counts within the cardiac ROI by the average number of counts within the mediastinal ROI. Calculation of WR was performed using the following formula (no correction for background): WR= (HMR_early_ − HMR_late_)/(HMR_early_) × 100%.^15^

### RDN Procedure

Pre-procedurally, 100IU heparin/kg was administered to achieve an active clotting time > 250 seconds. All procedures were performed under conscious sedation. After administration of local anesthesia, common femoral artery access was achieved by an ultrasound-guided puncture and a 7-Fr sheath was then introduced. A 7-Fr guiding catheter was used to accommodate the Paradise^TM^ RDN catheter (ReCor Medical, Palo Alto, CA).^16^ After smoothly engaging the renal arteries by using a no-touch technique with the help of a standard high-torque BHW coronary guidewire, selective renal artery angiograms were made. The Paradise^TM^ system ablation catheter was then advanced over the BHW wire. The Paradise^TM^ catheter has a distal balloon which is pressurized by the Paradise system to a range of 1.5 to 2.0 ATM using sterile circulating water. The ultrasound transducer is located within the balloon (balloon diameters 5 to 8 mm). A total of 2 to 3 ultrasound emissions of 6 to 10 seconds each were delivered per artery.

### Statistical Analysis

Continuous variables were expressed as mean ± standard deviation (SD). Continuous variables were compared using Student’s t test. Categorical variables were expressed as percentages and were compared using the *χ*^2^ test or Fisher’s Exact test when appropriate. Early and late HMR and WR were compared using the Wilcoxon signed-rank test. All statistical tests are 2-tailed. A *P* value < 0.05 was considered statistically significant. Statistical analysis was performed using SPSS statistical analysis (version 22.0).

## Results

### Study Population

Mean age of the patients was 57 ± 11 years, 90% were male, and 90% were in CCS class III or IV. None of the patients had diabetes, and hypertension was present in 80% of the cases.

All patients were using anti-anginal therapy using long-acting nitrates (10/10) and calcium channel blockers (8/10). Most patients had a history of prior coronary revascularization (Table 1).

**Table 1:** Baseline characteristics

Total study population N=10	
Age, years	57 ± 11
Male n, (%)	9 (90)
BMI, kg/m^2^	26.6 ± 5.0
eGFR, mL/min	76 ± 17
Cardiovascular risk factors (%)	
Diabetes	0 (0)
Hypertension	8 (80)
Dyslipidemia	9 (90)
Smoker, current	3 (30)
Family history of premature CVD	7 (70)
Cardiovascular history (%)	
Prior MI	7 (70)
Prior PCI	8 (80)
24h ABPM, mmHg	121 ± 16/72 ± 8
Office BP, mmHg	143 ± 19/80 ± 10
Heart rate, bpm	62 ± 8
Angina-grading scale	
CCSI	–
CCSII	1 (10)
CCSIII	8 (80)
CCSIV	1 (10)
Echocardiographic parameters	
LVEF, %	59 ± 9.6
LVEDD, mm	50 ± 6.1
LVESD, mm	34 ± 4.9
Pharmacological therapy, n (%)	
Nitrates,	10 (100)
Calcium channel blockers^a^	8 (80)^a^
Selective beta-blockers	5 (50)
ACE/ATII	8 (80)
Aspirin	10 (100)
Diuretics	4 (40)
Statins	10 (100)

Variables are presented in mean ± SD or %

*ABPM*, ambulatory blood pressure measurement; *BP*, blood pressure; *BMI*, body mass index; *CCS*, Canadian cardiovascular society grading of angina pectoris; *CVD*, cardiovascular disease; *eGFR*, estimated glomerular filtration rate; *MI*, myocardial infarction; *LVEF*, left ventricular ejection fraction; *LVEDD*, left ventricular end-diastolic diameter; *LVESD*, left ventricular end-systolic diameter; *PCI*, percutaneous coronary intervention

^a^N=2 were intolerant for calcium channel blockers

### Primary Efficacy Endpoint

#### ^123^I-MIBG change

No significant change was observed in early HMR (pre-RDN: 2.74 [2.10 to 3.21] vs 6 months post-RDN: 2.57 [2.20 to 3.00]; *P* = 0.76) and in late HMR (pre-RDN: 2.56 [2.18 to 3.20] vs 6 months post-RDN: 2.36 [2.13 to 3.22]; *P* = 0.22). No significant change was observed in WR (pre-RDN: 15.0 [10.5 to 18.5] vs 6 months post-RDN: 13.0 [6.0 to 22.0]; *P* = 0.22) (Table 2).

**Table 2:** Cardiac sympathetic nerve activity measured by ^123^I-MIBG pre- and 6 months post-procedure, expressed in median [IQR]

	Pre-RDN	6 months	*P*
Early HMR	2.74 [2.10–3.21]	2.57 [2.20–3.00]	0.76
Late HMR	2.56 [2.18–3.20]	2.36 [2.13–3.22]	0.22
WR	15.0 [10.5–18.5]	13.0 [6.0–22.0]	0.22

Variables are presented in mean ± SD

*HMR*, heart/mediastinum ratio; *WR*, washout rate

### Primary Safety Endpoints

There were no peri-procedural complications. No adverse events including death, stroke, or renal artery stenosis occurred during 6-month follow-up. Renal function remained unchanged, and estimated glomerular filtration rate (eGFR) was 76 ± 17 mL/min pre-RDN vs 75 ± 18 mL/min at 6-month follow-up (*P* = 0.68).

### Secondary Endpoints

#### Outcome on angina and quality of life

CCS class improved significantly from 3.00 ± 0.47 (pre-RDN) to 1.80 ± 0.92 at 6-month follow-up, *P* = 0.005.

**Figure 2:**
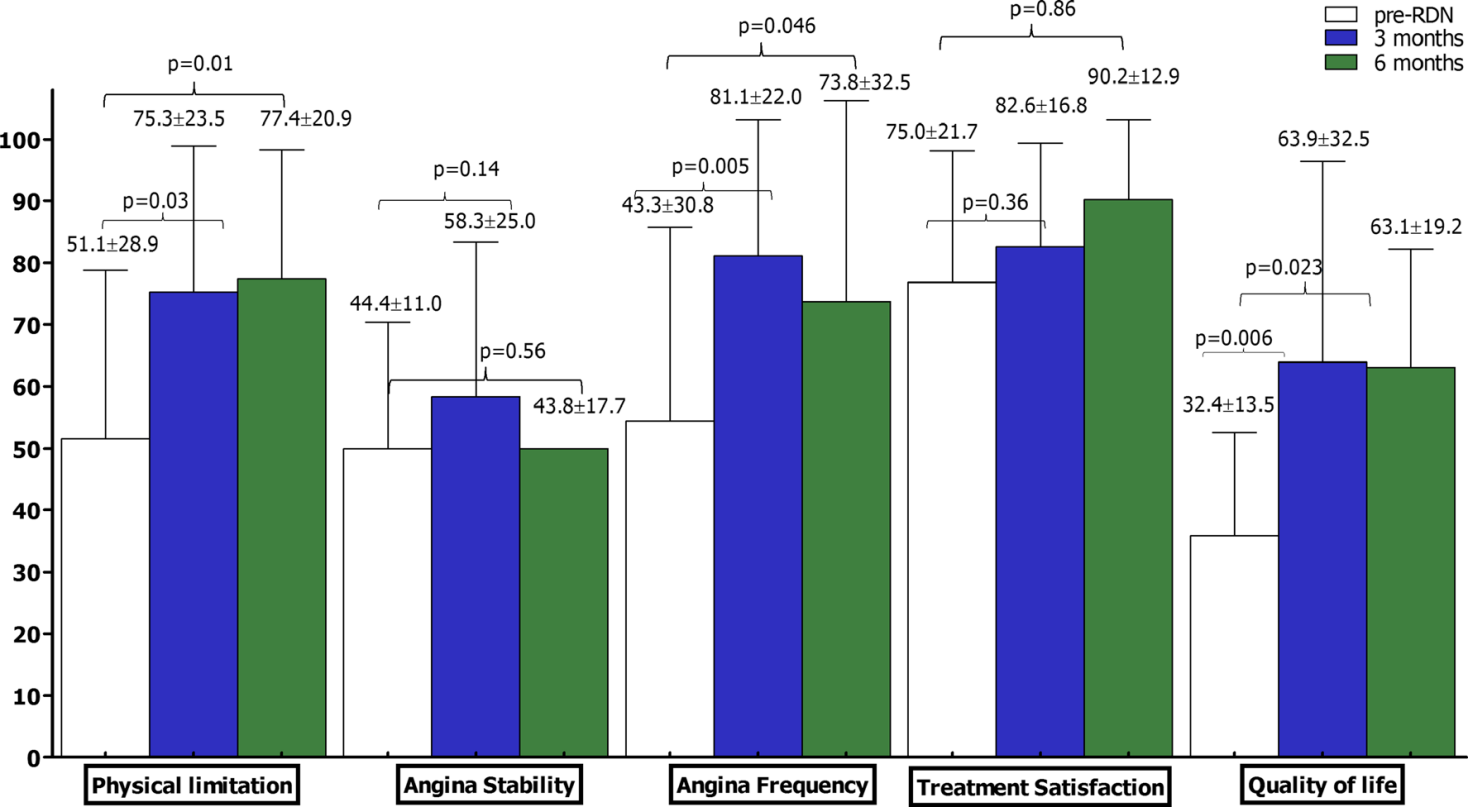
Seattle Angina Questionnaire (SAQ) SAQ scale (each scale is a score of 0 to 100, wherein higher scores indicate better function or less angina/limitation and better quality of life)

The SAQ results showed significant improvements in 3/5 subscales at 3- and 6-month follow-up; patients were less limited in daily activities due to angina; angina frequency decreased significantly and quality of life improved as compared to pre-RDN. Angina stability and treatment satisfaction remained unchanged at 3- and 6-month follow-up (Figure 2).

#### Blood pressure change

A numerical decrease in both office- and ambulatory BP at 6 months was found as compared to pre-RDN. Office BP changed from 143 ± 19/80 ± 10 mmHg pre-RDN to 132 ± 12/77 ± 7 mmHg at 6-month follow-up and 24h ABPM decreased from 121 ± 16/72 ± 8mmHg to 112 ± 8/70 ± 7 mmHg (*P* = ns for all).

#### Change in medication

**Figure 3:**
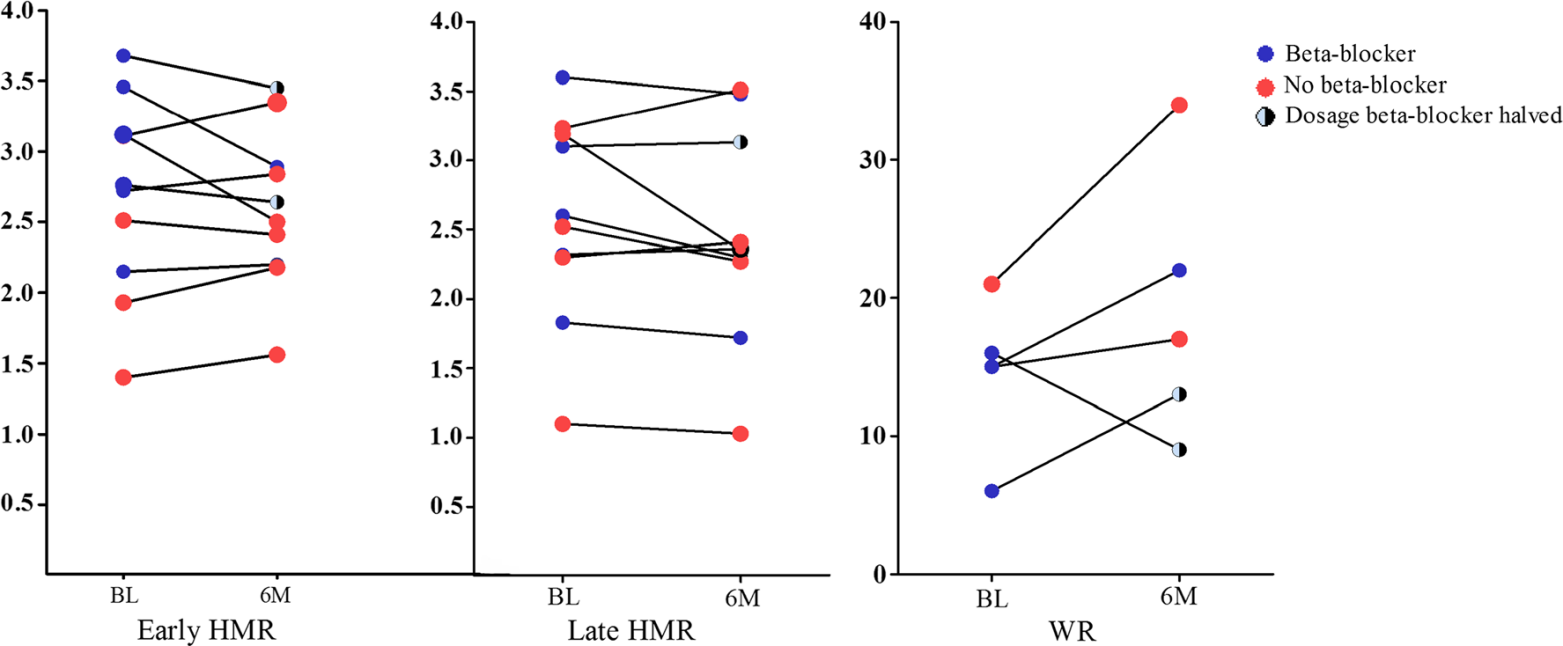
Change in early and late heart-to-mediastinum ratio (HMR), and washout rates (WR) at 6-month follow-up. Legend: 5 patients were off beta-blocker (red bullet) during the course of the study; 2 patients were on beta-blockers (same dose) at baseline and follow-up; 3 patients had beta-blockers at baseline and their dosage was halved (N = 2) and N = 1 was stopped at 6 months. In 5 patients, no washout rate (WR) could be calculated at baseline or 6 months

During the course of the study, long-acting nitrates and calcium channel blockers were decreased or stopped in 4 patients, while dosages were increased in 3 patients (Figure 3).

## Discussion

This present single-center pilot study demonstrated that RDN might significantly improve quality of life at 6 months post-procedure. However, no significant change was observed in cardiac sympathetic nerve activity as assessed by ^123^I-MIBG at 6 months post-procedure.

The autonomic nervous system is a key regulator of coronary vasomotion and an imbalance between the sympathetic and parasympathetic nervous system has been reported in patients with VSA.^4^,^17^,^18^ Provocation testing with parasympathetic agents such as acetylcholine or ergonovine can be performed in an attempt to confirm the diagnosis.^19^ In vitro, sympathetic agents like norepinephrine were used to test the severity of vasoconstriction or spasm.^20^

In the present study, we directly assessed cardiac sympathetic nerve activity by ^123^I-MIBG imaging, in line with previous work demonstrating the potential use of the technique in diagnosing VSA.^21^^123^I-MIBG is a physiologic analog of norepinephrine and acts selectively on sympathetic nerve endings. By using cardiac neurotransmission imaging, global information about neuronal function can be expressed in early, but more specifically in late HMR (reflecting the storage regional distribution and release of ^123^I-MIBG); additionally, the WR reflects the neuronal integrity or sympathetic tone.^14^^123^I-MIBG uptake is quantified by calculating an HMR after drawing regions of interest over the heart and mediastinum. Normal values for HMR and WR are 2.5 ± 0.3 or greater and 20 ± 10% or less, respectively.^22^ Little data are available on reference values in patients with VSA. Taki et al. reported higher WR in patients with VSA, probably due to increased sympathetic tone resulting in increased turnover of MIBG or impaired MIBG reuptake at the nerve endings.^23^,^24^ In addition, Arbab et al. showed a late HMR of 1.80 ± 0.60 in nine patients with VSA which was numerically lower as compared to healthy controls (2.00 ± 0.36).^25^ In the present study, pre-RDN HMR and WR were in line with previously published reference values for healthy control groups. Even in the cohort of patients with positive methylergonovine testing, mean late HMR was within the normal range. Our observations question whether HMR and WR in patients with VSA are truly significantly different than in healthy controls. Of note, several minor differences in imaging acquisition between this and other studies should be acknowledged such as the use of different collimators and radioactive compounds.

RDN has been studied since 2008 for its potential to control blood pressure in patients with therapy resistant hypertension, in which increased SNS activity has been hypothesized to play an important role.^26^ Only very recently, the results of several sham-controlled randomized trials demonstrated that the therapy, conducted using a radiofrequency ablation catheter, might significantly decrease blood pressure.^27^ At the same time, a series of studies were conducted to assess potential pleiotropic effects of RDN.^28^,^29^ In the present study, we attempted to further extend these findings in a cohort of VSA patients without residual regular therapeutic options. Given the limited options in objectively assessing the severity of signs and symptoms of these patients, we decided to further study the effect of RDN by using ^123^I-MIBG imaging. We hypothesized that post-RDN, late HMR would increase and WR would decrease as compared to pre-RDN.^23^,^25^ However, 6 months post-RDN, we found no meaningful differences in both early and late HMR and WR. The latter thus again questions the role of the SNS in patients with VSA and the potential of RDN to decrease intra-cardiac sympathetic nerve activity.

Nevertheless, we found significant improvements in angina class and quality of life at 6 months along with a numerical decrease in BP. Following treatment with the Paradise ultrasound balloon catheter, mean systolic ambulatory blood pressure decreased with 9mmHg at 6 months. Although these figures did not reach statistical significance in the present small single-center study, the primary results of the RADIANCE SOLO trial assessing the safety and efficacy of the same device in a multicenter randomized sham-controlled setting showed a significant decrease in daytime 24h ABPM as compared to sham.^30^

## Limitations

There are several limitations that should be taken into account. First, results were based on a small pilot study (N = 10) and should be considered hypothesis generating. Using the results of the present study along with the findings of previous work by Arbab et al, a total of 70 patients would be needed to support that RDN results in a 20% increase in late HMR. Second, WR was calculated using one of the methods as described by Flotats et al.,^31^ in which we used the actual heart counts for calculating WR, instead of ratios. Third, while the positive finding of a significant reduction in angina class could be interpreted as promising, a potential placebo effect of the treatment cannot be ruled out. In the recently published sham-controlled ORBITA trial, a sham procedure was able to significantly improve at least 3 subscales of the SAQ in patients with stable angina and significant coronary artery disease.^32^ Fourth, anti-anginal drug regimen was changed in 7/10 of the patients [including an increase in nitrates (n = 1) and diltiazem (n = 1)]. The impact of medication on MIBG uptake should be taken into account. Jacobson et al. described that the MIBG uptake could be inhibited mostly by the use of beta-blockers such as labetalol. Less evidence is available on the MIBG-inhibitory effect by calcium channel blockers.^33^ Furthermore, we acknowledge the fact that half of the patients were still using selective beta-blockers that were deliberately continued mostly due to the presence of a myocardial infarction in the past; in patients (N = 3), beta-blockers were stopped or dosages changed during follow-up. However, also in patients in whom beta-blockers were either stopped or dosages were changed, no change in late HMR was found which was in line with results in patients without changes in drug regimen.

Finally, we cannot exclude the fact that apart from autonomic nervous system tone, multiple other alternative factors might play a role in triggering coronary spasms such as endothelial dysfunction or microvascular dysfunction.^34^,^35^

## New Knowledge Gained

The results in our study are promising and might fuel future discussion on the potential use of RDN in patients with vasospastic angina. Larger, randomized trials are needed to demonstrate the value of ^123^I-MIBG imaging post-RDN in patients with VSA.

## Conclusion

RDN resulted in significant improvements in angina class and quality of life at 6 months in patients with refractory vasospastic angina. RDN did not result in significant changes in cardiac sympathetic nerve activity as measured using ^123^I-MIBG.

## Electronic supplementary material

Below is the link to the electronic supplementary material.

Supplementary material 1 (PPTX 1124 kb)

## Abbreviations

24h ABPM: 24h ambulatory blood pressure measurement
CT: Computed tomography
HMR: Heart-to-mediastinum ratio
^123^I-MIBG: Iodine-123 labeled meta-iodobenzylguanidine
MRI: Magnetic resonance imaging
MBq: Megabecquerel
RDN: Renal sympathetic denervation
SNS: Sympathetic nervous system
VSA: Vasospastic angina
WR: Washout rates
