# Supplementary material for: Renal sympathetic denervation in patients with vasospastic angina
Source: J Nucl Cardiol. 2019 Feb 13;27(6):2202–9. doi: 10.1007/s12350-019-01598-y (PMC7749092; doi:10.1007/s12350-019-01598-y)
Supplement: Supplementary file 1 — Supplementary material 1 (PPTX 1124 kb) [file 12350_2019_1598_MOESM1_ESM.pptx]

## Slide 1
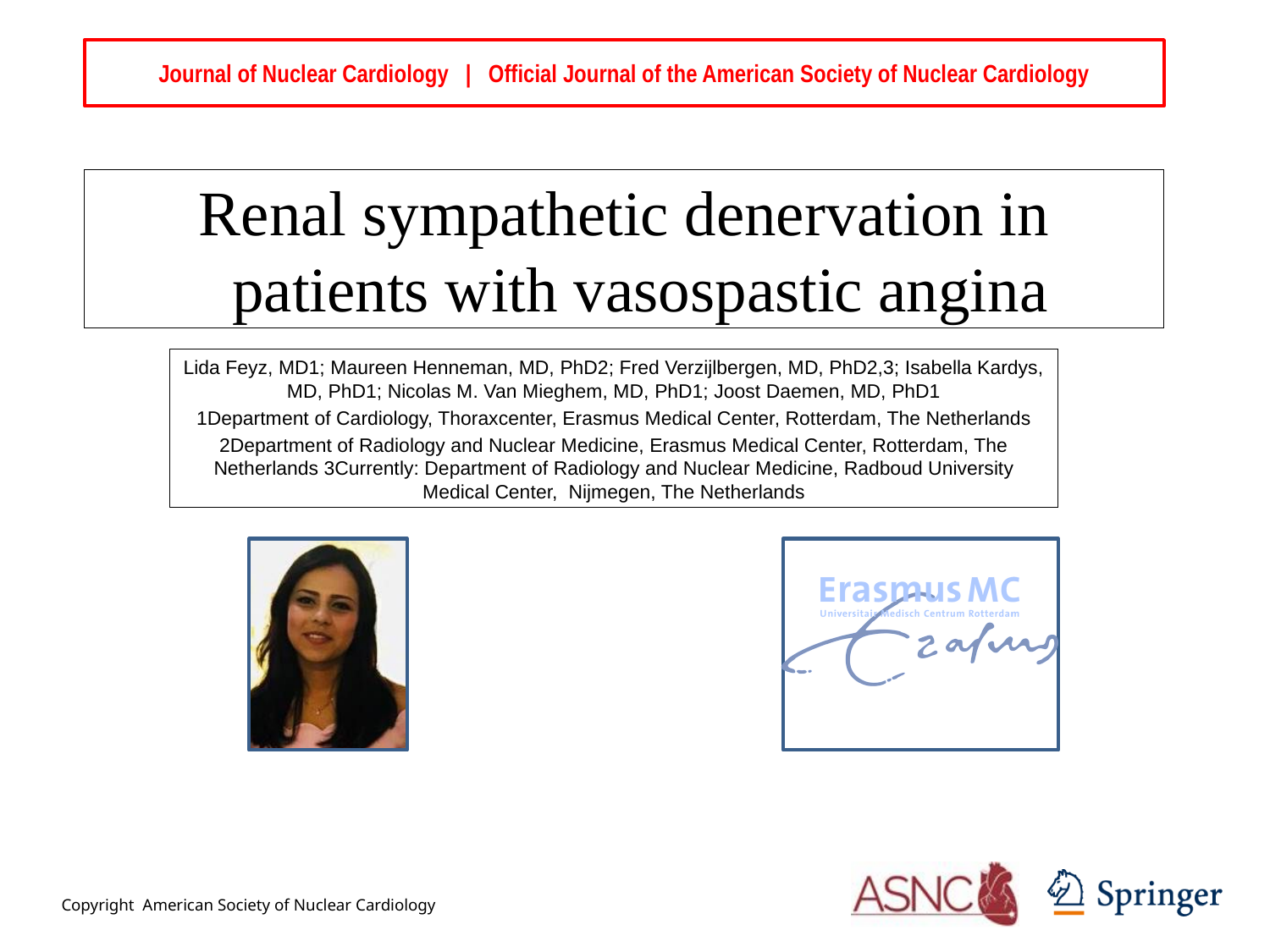

Journal of Nuclear Cardiology | Official Journal of the American Society of Nuclear Cardiology
# Renal sympathetic denervation in patients with vasospastic angina
Lida Feyz, MD1; Maureen Henneman, MD, PhD2; Fred Verzijlbergen, MD, PhD2,3; Isabella Kardys, MD, PhD1; Nicolas M. Van Mieghem, MD, PhD1; Joost Daemen, MD, PhD1
1Department of Cardiology, Thoraxcenter, Erasmus Medical Center, Rotterdam, The Netherlands
2Department of Radiology and Nuclear Medicine, Erasmus Medical Center, Rotterdam, The Netherlands 3Currently: Department of Radiology and Nuclear Medicine, Radboud University Medical Center, Nijmegen, The Netherlands
Copyright American Society of Nuclear Cardiology

## Slide 2
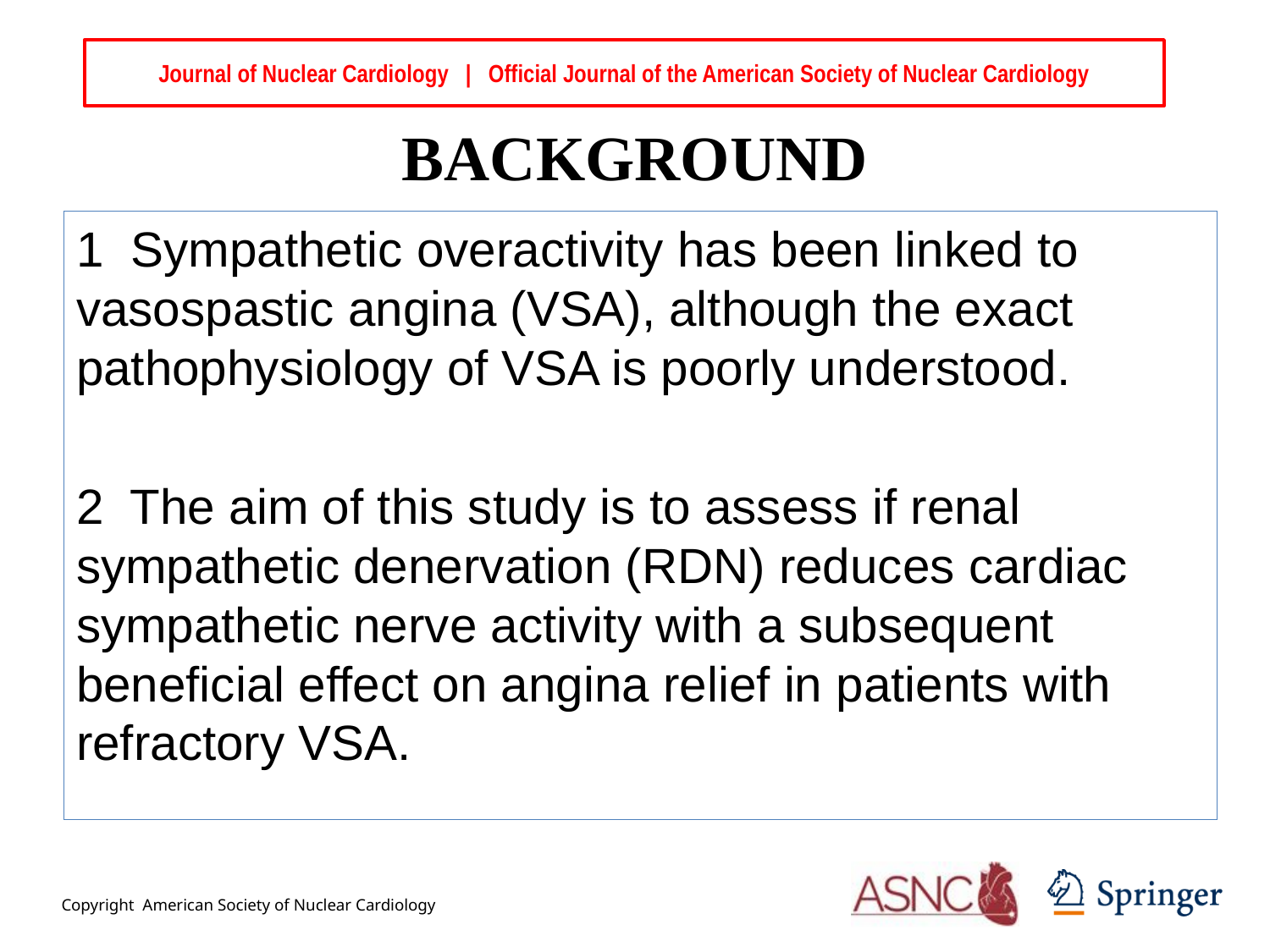

Journal of Nuclear Cardiology | Official Journal of the American Society of Nuclear Cardiology
# BACKGROUND
1 Sympathetic overactivity has been linked to vasospastic angina (VSA), although the exact pathophysiology of VSA is poorly understood.
2 The aim of this study is to assess if renal sympathetic denervation (RDN) reduces cardiac sympathetic nerve activity with a subsequent beneficial effect on angina relief in patients with refractory VSA.
Copyright American Society of Nuclear Cardiology

## Slide 3
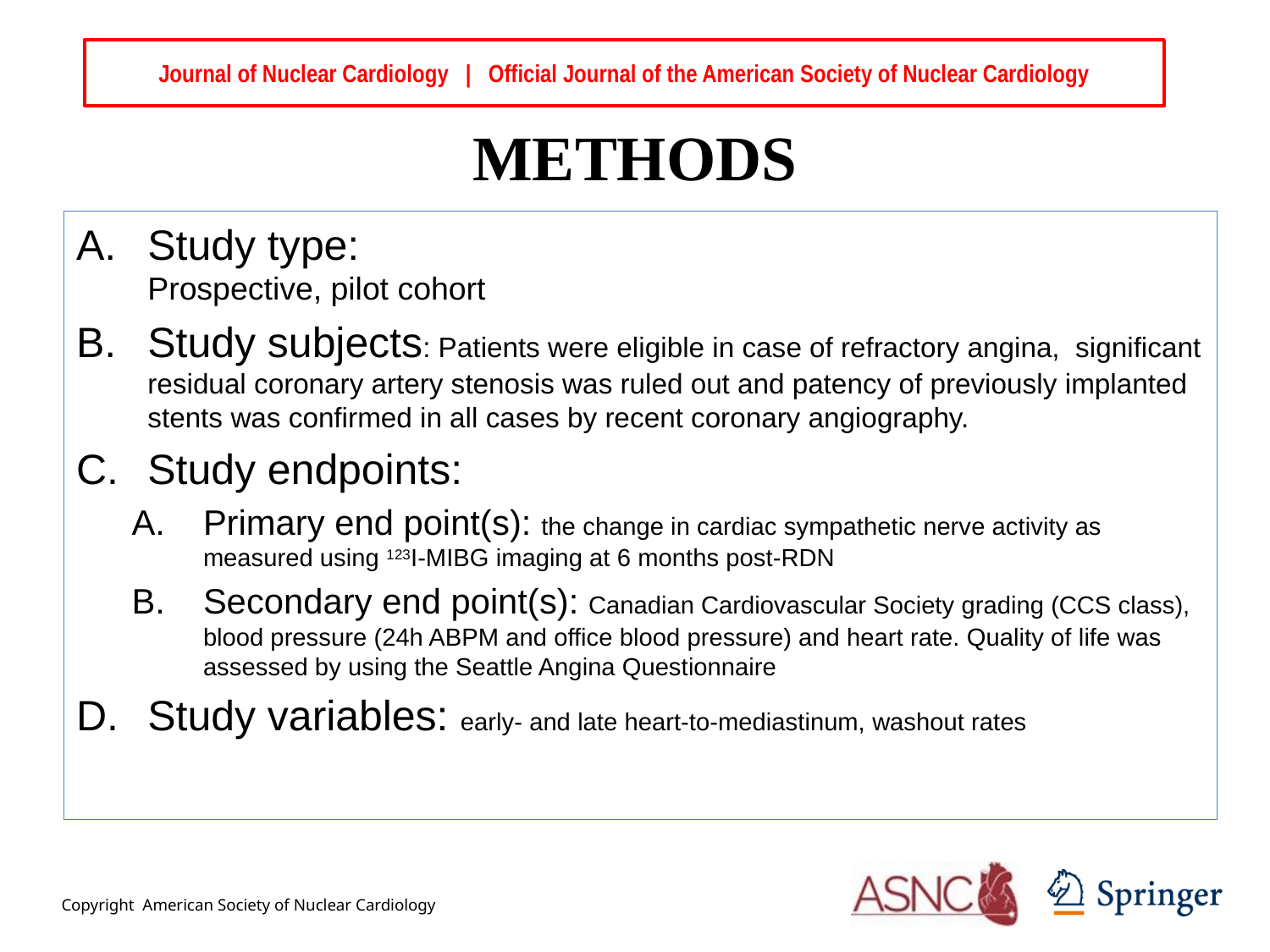

Journal of Nuclear Cardiology | Official Journal of the American Society of Nuclear Cardiology
# METHODS
Study type:
Prospective, pilot cohort
Study subjects: Patients were eligible in case of refractory angina, significant residual coronary artery stenosis was ruled out and patency of previously implanted stents was confirmed in all cases by recent coronary angiography.
Study endpoints:
Primary end point(s): the change in cardiac sympathetic nerve activity as measured using 123I-MIBG imaging at 6 months post-RDN
Secondary end point(s): Canadian Cardiovascular Society grading (CCS class), blood pressure (24h ABPM and office blood pressure) and heart rate. Quality of life was assessed by using the Seattle Angina Questionnaire
Study variables: early- and late heart-to-mediastinum, washout rates
Copyright American Society of Nuclear Cardiology

## Slide 4
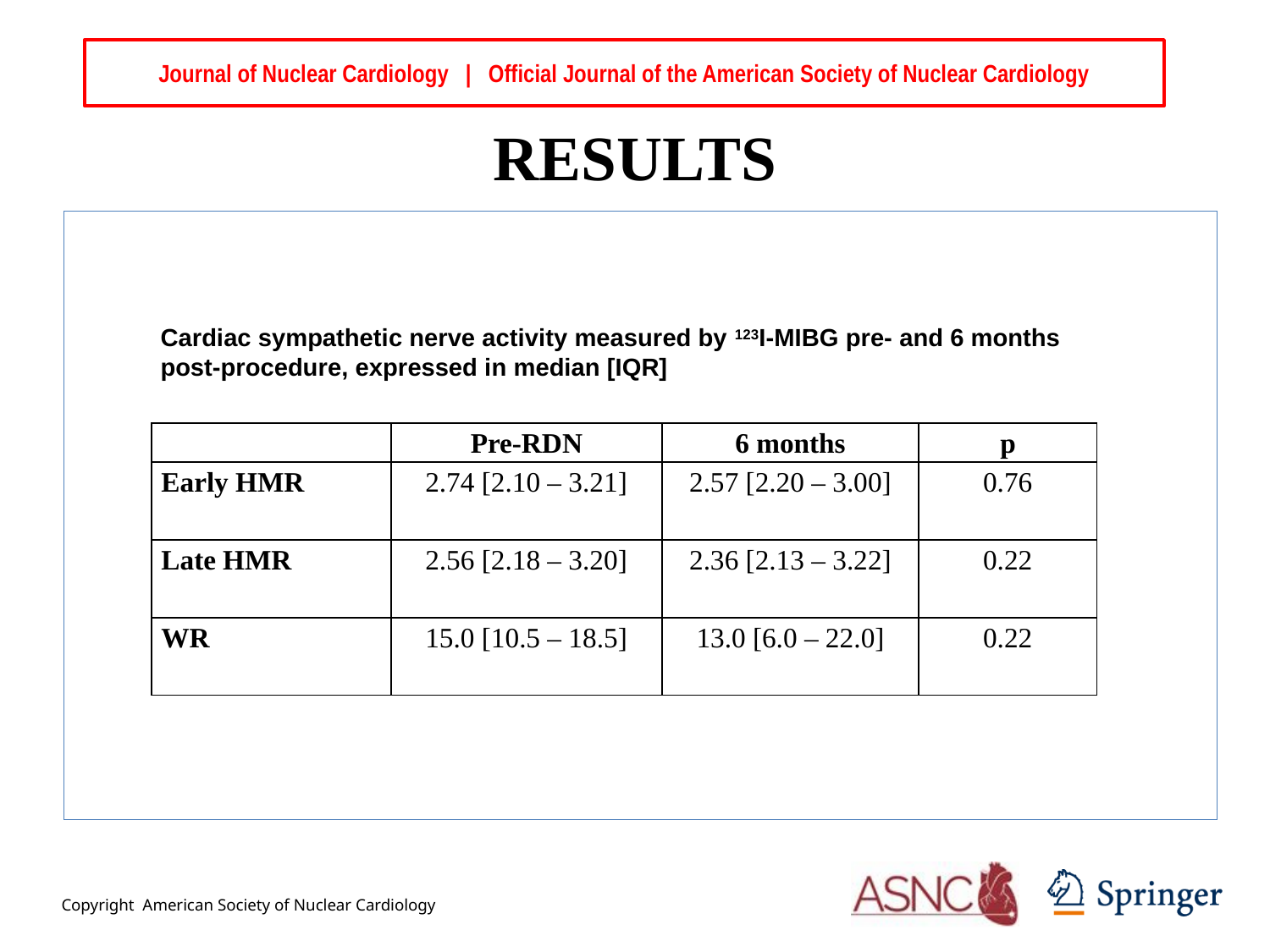

Journal of Nuclear Cardiology | Official Journal of the American Society of Nuclear Cardiology
# RESULTS
Cardiac sympathetic nerve activity measured by 123I-MIBG pre- and 6 months post-procedure, expressed in median [IQR]
| | Pre-RDN | 6 months | p |
| --- | --- | --- | --- |
| Early HMR | 2.74 [2.10 – 3.21] | 2.57 [2.20 – 3.00] | 0.76 |
| Late HMR | 2.56 [2.18 – 3.20] | 2.36 [2.13 – 3.22] | 0.22 |
| WR | 15.0 [10.5 – 18.5] | 13.0 [6.0 – 22.0] | 0.22 |
Copyright American Society of Nuclear Cardiology

## Slide 5
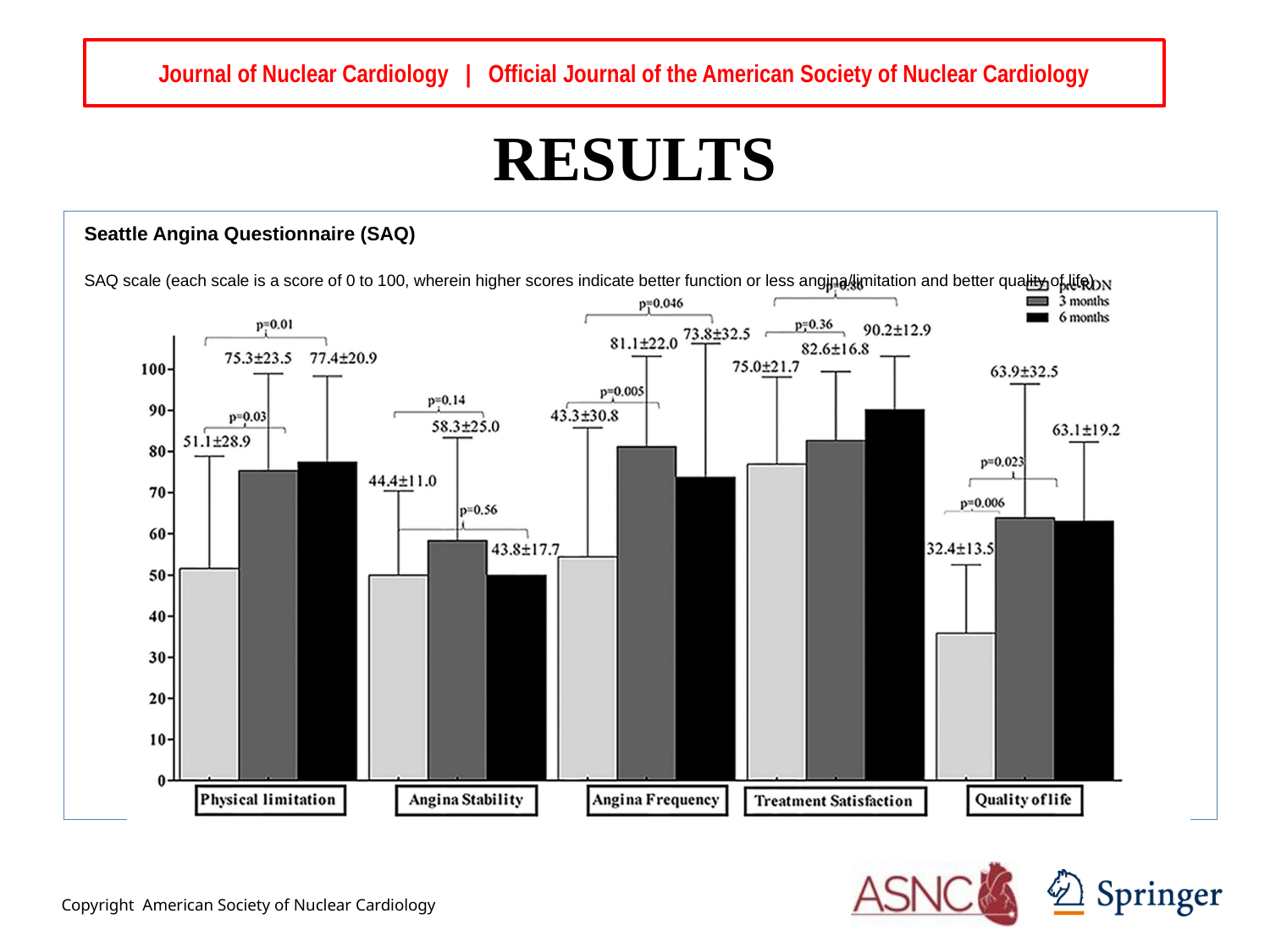

Journal of Nuclear Cardiology | Official Journal of the American Society of Nuclear Cardiology
# RESULTS
Seattle Angina Questionnaire (SAQ)
SAQ scale (each scale is a score of 0 to 100, wherein higher scores indicate better function or less angina/limitation and better quality of life)
Copyright American Society of Nuclear Cardiology

## Slide 6
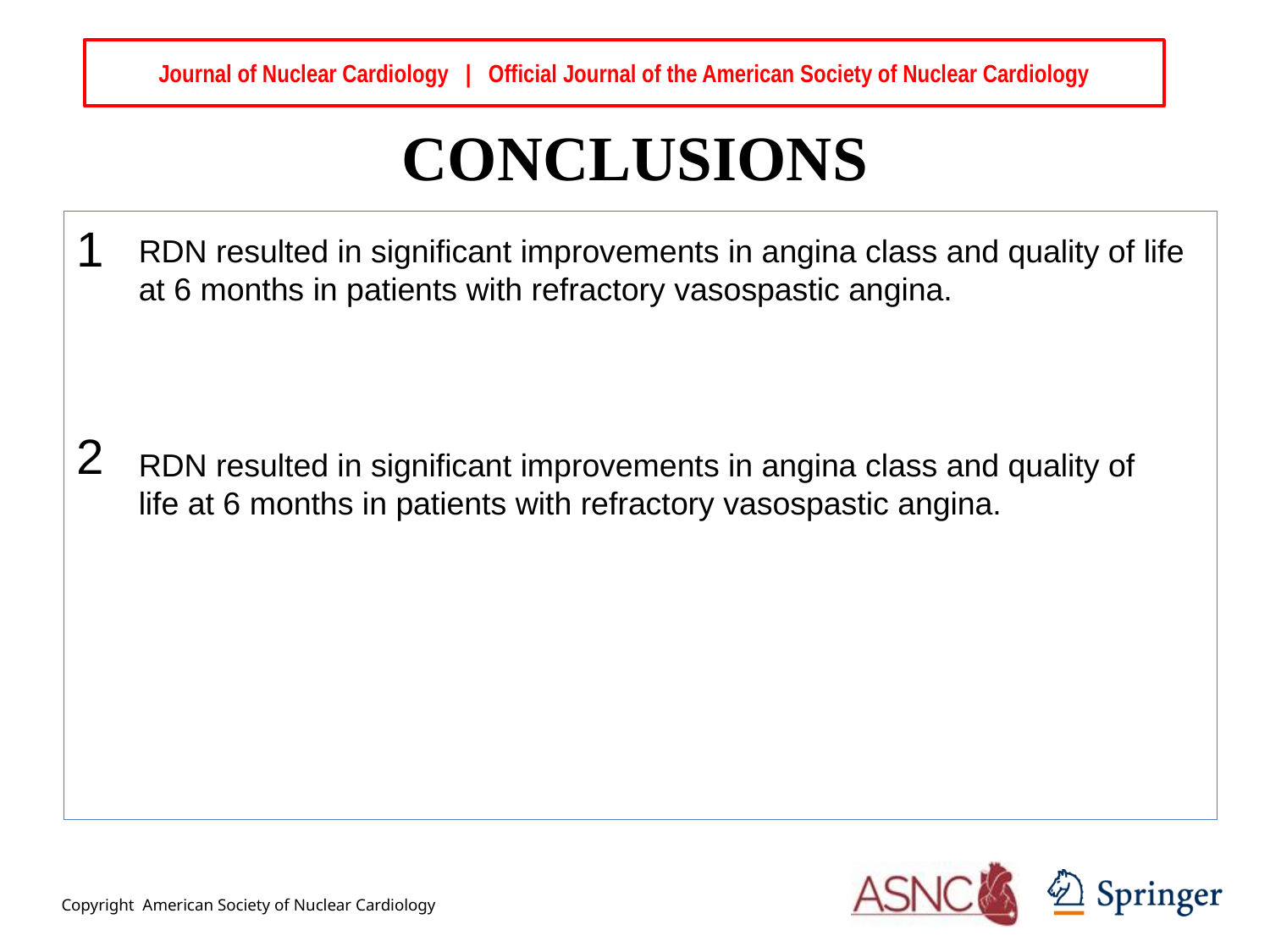

Journal of Nuclear Cardiology | Official Journal of the American Society of Nuclear Cardiology
# CONCLUSIONS
1
2
RDN resulted in significant improvements in angina class and quality of life at 6 months in patients with refractory vasospastic angina.
RDN resulted in significant improvements in angina class and quality of life at 6 months in patients with refractory vasospastic angina.
Copyright American Society of Nuclear Cardiology
